# MORE-Q, a dataset for molecular olfactorial receptor engineering by quantum mechanics

**DOI:** 10.1038/s41597-025-04616-6

**Published:** 2025-02-22

**Authors:** Li Chen, Leonardo Medrano Sandonas, Philipp Traber, Arezoo Dianat, Nina Tverdokhleb, Mattan Hurevich, Shlomo Yitzchaik, Rafael Gutierrez, Alexander Croy, Gianaurelio Cuniberti

**Affiliations:** 1 Institute for Materials Science and Max Bergmann Center for Biomaterials, TUD Dresden University of Technology, 01062 Dresden, Germany; 2 Institute of Physical Chemistry, Friedrich Schiller University Jena, 07737 Jena, Germany; 3 Institute of Chemistry and Center of Nanotechnology, The Hebrew University of Jerusalem, Jerusalem, 91904 Israel; 4 Dresden Center for Computational Materials Science (DCMS), TUD Dresden University of Technology, 01062 Dresden, Germany

**Keywords:** Sensors and biosensors, Biosensors, Chemical physics, Electronic structure

## Abstract

We introduce the MORE-Q dataset, a quantum-mechanical (QM) dataset encompassing the structural and electronic data of non-covalent molecular sensors formed by combining 18 mucin-derived olfactorial receptors with 102 body odor volatilome (BOV) molecules. To have a better understanding of their intra- and inter-molecular interactions, we have performed accurate QM calculations in different stages of the sensor design and, accordingly, MORE-Q splits into three subsets: i) MORE-Q-G1: QM data of 18 receptors and 102 BOV molecules, ii) MORE-Q-G2: QM data of 23,838 BOV-receptor configurations, and iii) MORE-Q-G3: QM data of 1,836 BOV-receptor-graphene systems. Each subset involves geometries optimized using GFN2-xTB with D4 dispersion correction and up to 39 physicochemical properties, including global and local properties as well as binding features, all computed at the tightly converged PBE+D3 level of theory. By addressing BOV-receptor-graphene systems from a QM perspective, MORE-Q can serve as a benchmark dataset for state-of-the-art machine learning methods developed to predict binding features. This, in turn, can provide valuable insights for developing the next-generation mucin-derived olfactory receptor sensing devices.

## Background & Summary

### Introduction

Nowadays, the increasing progress in artificial intelligence (AI) has boosted the development of AI-based technologies for the recognition of objects, faces, voices, and touch^1^. However, a significant gap remains in technologies capable of interpreting and predicting the chemical environment around us. In this sense, tailored electronic noses have recently emerged and are already capable of detecting, for example, volatile organic compounds (VOCs)^2,3^. The detection of VOCs emitted from the human body^4–6^, also known as body odor volatilomes (BOVs), can be used as characteristic fingerprints and have significant applications in healthcare^7^. The constituents of BOVs generally indicate the metabolic state of a person and are promising candidates for medical biomarkers in diagnosing a range of diseases^5,8–13^, *e.g*. Alzheimer^14,15^ and Parkinson^14–17^. A recent report documented that a ’super smeller’ could detect and distinguish the BOVs associated with Parkinson’s disease, emitted from sebum, from those of normal skin^18^ indicating the powerful body odor perception ability enabled by the olfactory system. Given these facts, the demand for fast and robust sensing materials for detecting BOV molecules remains consistently strong, particularly in medical diagnostics.

It is known that odor perception begins in the nose, where tens of thousands of odorants can be detected by the olfactorial receptors, which are composed of a glycoprotein layer (mucin) that covers the epithelium of olfactory and respiratory systems^19–21^. While there have been considerable efforts in investigating single odor (BOV) molecules^22–30^ and molecular receptors^31–34^, less information is available to accurately describe the physical and chemical interactions in BOV-receptor systems. Characterizing these interactions will deepen our understanding of the biomimetic olfactory system, paving the way for the rational design of receptors tailored for sensing applications^35^. To address this challenge, initial datasets of BOV-receptor systems have been developed^36–40^ (see Table 1). For instance, Mainland *et al*.^38^ provided the *in vitro* response of 73 odorants against a clone library of 511 human olfactorial receptors. Sharma *et al*.^39^ also developed the online platform OlfactionBase, which provides chemoinformatic properties (*e.g*. drug-likeness, pharmacokinetic profile, molecular weight, $\log P$) and odorant-receptor match information for 875 systems. In a more recent study, Lalis *et al*.^40^ developed M2OR database of 51,395 odorant-receptor systems for understanding the molecular mechanisms of olfaction. Numerous similar datasets have been published regarding odorant-receptor interactions and match information^37,41–43^. However, to the best of our knowledge, none of these datasets account for a quantum-mechanical (QM) treatment of physical and chemical interactions in odorant-receptor systems. This is of great relevance because accurately describing intermolecular interactions – such as van der Waals forces, hydrogen bonds, and dipole-dipole interactions – is essential for determining the correct docking configuration and binding features. Indeed, recent efforts have focused on developing QM datasets to understand the structure-property and property-property relationships in both small and large molecular systems^44–49^. Additionally, a few QM datasets for small molecular dimers have been generated, where the relevant property is solely the interaction energy^50–53^. Another open challenge in this field is the accurate investigation of the interaction of odorant-receptor systems on a substrate. Carbon-based materials such as graphene are promising sensing materials due to their high charge mobility and favorable surface-to-volume ratio^54–58^. Hence, it is essential to have a dataset that provides a QM description of both the structural and electronic properties of odorant-receptor systems, as well as their interaction with a sensing material.

**Table 1:** Main characteristics of recent publicly available olfactory receptor datasets.

Dataset	Receptor source	Total dimer configurations	QM properties	Surface interaction
Mainland *et al*.^38^	Human-cloned	37,303	No	No
OlfactionBase^39^	Human/mouse	875	No	No
M2OR^40^	Mammals	51, 415	No	No
OlfactionDB^37^	Human/mouse	~400	No	No
MORE-Q-G2	Mucin-derived	23,838	Yes	No
MORE-Q-G3	Mucin-derived	1,836	Yes	Yes

Note that among these, the MORE-Q dataset uniquely includes quantum-mechanical (QM) properties of the systems under study and is the only dataset that considers the interaction of BOV-receptor systems with a surface.

**Fig. 1:**
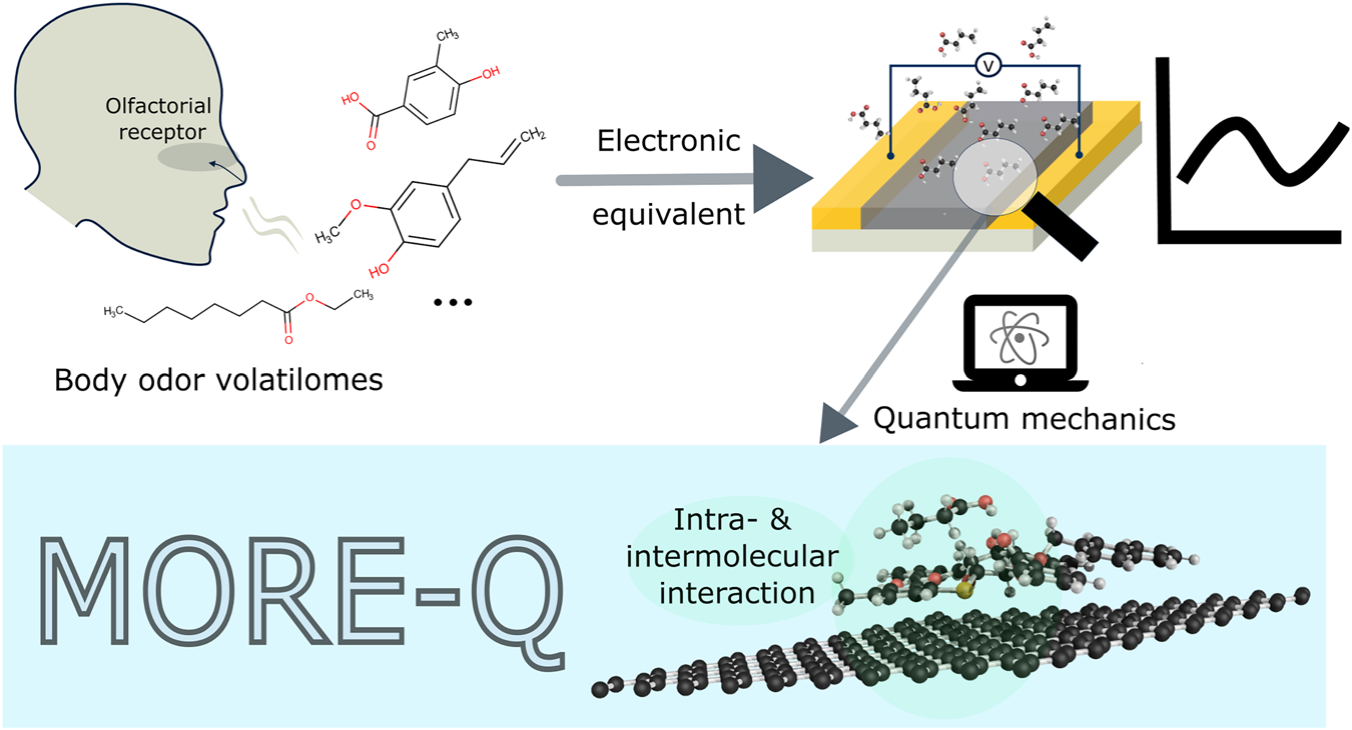
Graphical representation of the motivation for developing MORE-Q dataset (**M**olecular **O**lfactorial **R**eceptor **E**ngineering by **Q**uantum mechanics). Bio-electronic noses (top right panel) are designed as an electronic equivalent to the olfactory system (top left panel), *e.g*. for sensing body odor volatilomes (BOV). The MORE-Q dataset offers a comprehensive collection of quantum-mechanical properties and structural data that accurately describe intra- and intermolecular interactions in molecular sensors, see lower panel.

**Fig. 2:**
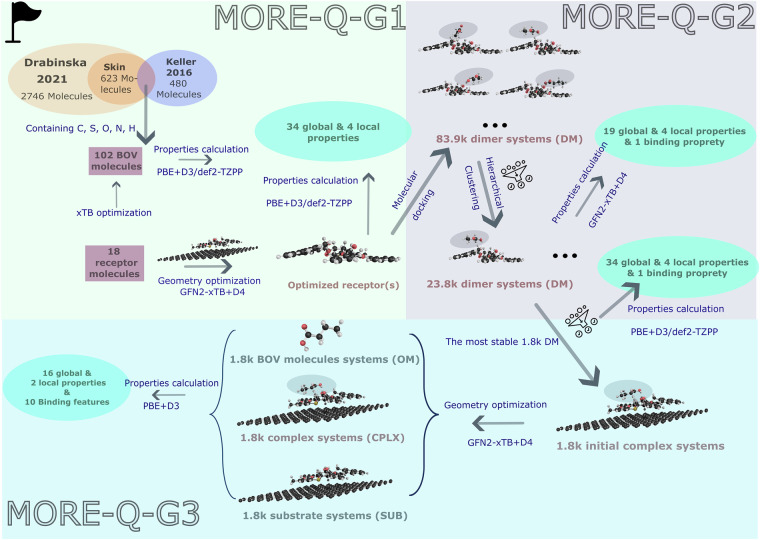
Schematic description of generation procedure of the MORE-Q dataset. MORE-Q is split into three subsets depending on the generation stage: MORE-Q-G1, MORE-Q-G2, MORE-Q-G3. In MORE-Q-G1, We first established a skin body odor set with 102 molecules from the intersection of two body odor resources such as those presented in Drabinska *et al*.^6^ and Keller *et al*.^64^. The structure of isolated BOV molecules and 18 receptor-graphene systems were optimized using GFN2-xTB with D4 dispersion correction. Physicochemical properties were posteriorly computed at the tightly converged PBE+D3 level of theory. For generating MORE-Q-G2, the conformational search of the BOV-receptor systems from MORE-Q-G1 was carried out using the docking program aISS code which employs xTB-IFF^72^ force field and a follow-up genetic algorithm, resulting in a total of 83,916 BOV-receptor dimer configurations. Following an RMSD-based hierarchical clustering method, 23,838 non-redundant configurations were selected and their properties were calculated at GFN-xTB+D4 theory level. The most energy-favorable 1,836 configurations were further selected, and their corresponding dimer properties were calculated both at the higher PBE+D3 theory level. In MORE-Q-G3, the selected 1,836 configurations from MORE-Q-G2 were optimized back on the graphene surface, forming the complex BOV-receptor-graphene systems (CPLX). The substrate (SUB) and BOV molecule (OM) systems were constructed by removing the BOV molecules and receptor+graphene, respectively. Next, PBE+D3 level simulations were conducted on the geometries of 1,836 CPLX, SUB, and OM systems, obtaining global and local properties, as well as the binding features of these systems. See “Methods" for more details.

To address these challenges, we introduce the MORE-Q dataset, which provides an extensive set of QM properties to accurately investigate the BOV-Receptor systems on a graphene surface, see Fig. 1. We have modeled 18 mucin-derived receptors and their combinations with 102 relevant skin BOV molecules^6^—both systems containing heavy atoms C, N, O, and S. The number of atoms of the receptor molecules ranges from 37 to 102 atoms, while for BOV molecules varies from 7 to 53 atoms. We have followed an exhaustive and systematic procedure to generate the final complex systems (*i.e*. BOV-receptor-graphene system) and compute the QM properties, resulting in the creation of three MORE-Q subsets: i) MORE-Q-G1 contains the QM data of isolated single BOV and receptor molecules, ii) MORE-Q-G2 contains the QM data of diverse configurations of the BOV-receptor systems, and iii) MORE-Q-G3 contains the QM data of dimers deposited on the graphene surface (see Fig. 2). The initial BOV and receptor molecules were optimized using the semi-empirical method GFN2-xTB that considers D4 dispersion correction^59^. Then, the configurations of the 1,836 BOV-receptor systems were screened by molecular docking using the automated Interaction Site Screening (aISS)^60^ submodule of the xTB code, applying the same level of theory as used in the geometry optimizations. Here, the hierarchical clustering method was used to filter similar geometries for each BOV-receptor combination, resulting in 23,838 dimer configurations. To generate the complex systems, we have considered the most energy-favorable dimer configuration for each unique combination (ranked by the interaction energy *E*_int_) and, then, deposited it on a graphene layer. Finally, each MORE-Q subset includes up to 39 physicochemical properties, encompassing global (molecular) and local (atom-in-a-molecule) properties, as well as binding features. All these properties were computed using the tightly converged PBE+D3 level of theory. As such, the MORE-Q dataset provides a comprehensive set of QM structural and property data for BOV-receptor and BOV-receptor-graphene systems, which can further enhance our understanding and the prediction of the performance of molecular sensors for digital olfaction.

### Key advancements

The MORE-Q dataset series aims to provide accurate QM data to gain insights into the interaction between BOV molecules and molecular receptors, as well as the effects of substrate deposition on binding features. To achieve this objective, we have ensured that MORE-Q includes the following attributes:

- The MORE-Q dataset comprises the QM structural and property data of 18 newly synthesized mucin-derived receptors^32,61,62^ and 102 skin molecules, carefully selected from an extensive pool of 2, 746 BOV molecules, representing a large swath of the chemical space of BOV molecules.
- We have exhaustively explored the potential energy surface (PES) of BOV-receptor systems by using the automated interaction site screening method in xTB code^60^, at the GFN2-xTB level of theory^59^ with D4 dispersion correction^63^.This procedure allowed us to determine the 1,836 most energetic-favorable BOV-receptor dimer configurations.
- The MORE-Q dataset provides extensive sets of QM global and local properties (up to 39) for single BOV/receptor molecules (MORE-Q-G1), molecular dimers (MORE-Q-G2), and complex systems (MORE-Q-G3), offering more comprehensive data compared to all other aforementioned BOV-receptor interaction datasets. These properties can be utilized in machine learning (ML) methods (*e.g*. as QM descriptors) to uncover and elucidate structure-property and property-property relationships between the building blocks, as well as the entire molecular sensor.
- The MORE-Q-G3 dataset involves complex electronic structure calculations, such as the determination of the work function (WF) *ϕ*, which can be related to the sensing response of molecular devices. This property offers a more realistic validation of the ML models trained on our QM data, intending to design novel molecular sensors.

## Methods

### BOV and receptor molecules

The selection of the initial set of BOV molecules dataset was based on two main works^6,64^. In the first one, Drabińska *et al*.^6^ reports a meta-analysis of the available literature on chemical substances that have been documented in the human body. Here, 2,752 molecules are categorized into feces, urine, breath, skin, milk, blood, saliva, and semen. Since a central aspect of digital olfaction is the perception of odor molecules, we have also examined the work done by Keller *et al*.^64^, and consider 480 BOV molecules with available perceptions from their dataset. Then, by intersecting both datasets, we ended up with 102 skin-related molecules that include the heavy elements C, S, O, and N (see Fig. 2). Their isomeric SMILE strings and the corresponding initial geometries were extracted from the large public molecular database PubChem^65^. The molecular size of the BOV molecules varies from 7 to 53 atoms. The two-dimensional chemical representation of these molecules has been plotted in Fig. S1 of the Supporting Information (SI). Furthermore, we have modeled newly-synthesised 18 bio-inspired (mucin-derived) receptors^32,61,62^ that are composed of glycan modified by aromatic decoration for surface adhesion. D-galactose, one of the most common glycans in the extracellular matrix, was used as a scaffold for aromatic decorated monosaccharide receptor’s library, which is obtained by a multistep chemical synthesis^62^ of monosaccharides. The synthetic approach provides the ability to install specific groups of various natures on the monosaccharide thereby enabling tuning the receptor’s affinity toward odorants. Using this ability we control the rigidity, hydrophobicity, and polarizability of glycan-based receptors. The molecular size of the receptors ranges from 37 to 102 atoms, including the heavy atoms C, N, O, and S. A detailed description and full characterization can be found in Refs. ^32,62^. We show the two-dimensional chemical structures of the 18 receptors in Fig. S2 of the SI.

### MORE-Q-G1 dataset generation: properties of monomers

The MORE-Q-G1 dataset contains the quantum-mechanical (QM) properties of the optimized structures of 102 BOV molecules and 18 molecular receptors. The structures of each BOV molecule were first optimized using the semi-empirical method GFN2-xTB that considers D4 dispersion correction as it is implemented in the xTB packages (version 6.6.0)^59^. A stringent convergence criterion for energies and gradient norms was set to 5 × 10^−8^ *E*_h_ and $5\times 1{0}^{-5}\,{E}_{h}\cdot {a}_{0}^{-1}$, respectively. For the molecular receptors, we deposited them directly on a 10 × 10 graphene layer containing 200 C atoms with the fixed vacuum layer *z* = 50.68 Å in a periodic simulation box. The geometry optimization of the receptor-graphene systems (referred to as the ’SUB’ system in other sections) was conducted using DFTB+ package^66,67^ and considering the GFN2-xTB Hamiltonian with D4 dispersion correction. The thresholds for SCC convergence and the maximal atomic force were set to 1 ⋅ 10^−5^ and $1\cdot 1{0}^{-4}\,{E}_{h}\cdot {a}_{0}^{-1}$, respectively. The Fermi smearing in the optimization was set to 300 K and simulation was conducted at Gamma point. During the optimization, lattice vector angles and slab thickness were fixed. For the initial adsorption of each receptor on graphene, configurations were set to have maximal *π* − *π* stacking between the pyrene rings of the receptors and the graphene layer. Notice that, instead of optimizing the receptors as an isolated system, we directly optimized them within the periodic graphene system to avoid self *π* − *π* stacking in the isolated state, which would impede stable adsorption on the graphene surface.

#### Calculation of physicochemical properties

We have computed 39 QM global and local properties of the optimized structures of BOV and receptor molecules, see Table 2. To do this, single-point calculations were conducted employing density-functional theory (DFT) at the PBE level with def2-TZVPP basis set and D3 dispersion correction, as implemented in the ORCA software (version 5.0.3)^68^. Energy components, orbital energies, *C*_6_ dispersion coefficient, atomic forces, dipole moment, and quadrupole moment were extracted from the output files of the self-consistency (SCF) calculations. The molecular isotropic polarizability and the polarizability tensor were analytically calculated through the coupled-perturbed SCF equations (CP-SCF). The radius of gyration was calculated per each structure by ${R}_{g}=\frac{\Sigma {m}_{i}\cdot {r}_{i}^{2}}{\Sigma {m}_{i}}$, where *m*_*i*_ and *r*_*i*_ are the *i*^th^ atom mass and the corresponding distance to the molecular center of mass, respectively. The atomic charges were computed by performing Mulliken^69^, Loewdin^70^ and Mayer^71^ population analysis.

**Table 2:** List of physicochemical properties of BOV and molecular receptors contained in MORE-Q-G1 subset. Each property presents a name, symbol, unit, dimension, type, and corresponding key in the HDF5 file.

#	Property	Symbol	Unit	Dimension	Type	HDF5 keys
1	Atomic numbers	—	—	N	A	‘atNUM’
2	Atomic positions	—	Å	3N	A	‘atXYZ’
3	Total PBE+D3 energy	*E*_tot_	eV	1	M	‘ePBE+D3’
4	Nuclear repulsion energy	*E*_nuc_	eV	1	M	‘eNUC’
5	Electronic repulsion energy	*E*_ele_	eV	1	M	‘eELE’
6	One electron energy	*E*_1e_	eV	1	M	‘e1E’
7	Two electron energy	*E*_2e_	eV	1	M	‘e2E’
8	Virial potential energy	*E*_pe_	eV	1	M	‘ePE’
9	Virial kinetic energy	*E*_ke_	eV	1	M	‘eKE’
10	Exchange energy	*E*_x_	eV	1	M	‘eX’
11	Correlation energy	*E*_c_	eV	1	M	‘eC’
12	Exchange-correlation energy	*E*_xc_	eV	1	M	‘eXC’
13	Total D3 energy	*E*_D3_	eV	1	M	‘eD3’
14	Dispersion E6 energy	*E*_6_	eV	1	M	‘eE6’
15	Dispersion E8 energy	*E*_8_	eV	1	M	‘eE8’
16	HOMO energy	*E*_HOMO_	eV	1	M	‘eH’
17	LUMO energy	*E*_LUMO_	eV	1	M	‘eL’
18	HOMO-LUMO gap	*E*_GAP_	eV	1	M	‘HLgap’
19	Orbital energies	*E*_oe_	eV	*	M	‘eORB’
20	Isotropic molecular *C*_6_ coefficient	*C*_6_	${E}_{{\rm{h}}}\cdot {a}_{0}^{6}$	1	M	‘mC6’
21	Electronic dipole moment	*μ*_elc_	D	3	M	‘vEDIP’
22	Nuclear dipole moment	*μ*_nuc_	D	3	M	‘vNDIP’
23	Total dipole moment	*μ*	D	3	M	‘vDIP’
24	Scalar total dipole moment	*μ*_s_	D	1	M	‘DIP’
25	Rotational spectrum constant	*B*	MHz	3	M	‘vRS’
26	Rotational dipole moment	*μ*_B_	d	3	M	‘vRSDIP’
27	Nuclear quadrupole moment tensor	*Q*_nuc_	$e\cdot {a}_{0}^{2}$	6	M	‘NQP’
28	Electronic quadrupole moment tensor	*Q*_ele_	$e\cdot {a}_{0}^{2}$	6	M	‘EQP’
29	Total quadrupole moment tensor	*Q*	Buckingham	6	M	‘TQP’
30	Isotropic molecular quadrupole	*Q*_s_	Buckingham	1	M	‘mQP’
31	Molecular polarizabillity tensor	*α*	${a}_{0}^{3}$	6	M	‘mTPOL’
32	Molecular isotropic polarizability	*α*_s_	${a}_{0}^{3}$	1	M	‘mPOL’
33	Radius of gyration	*R*_g_	Å	1	M	‘RG’
34	Inertia moment tensor	*I*_TS_	amu ⋅Å^2^	6	M	‘IM’
35	Mulliken atomic charge	*q*_mu_	*e*	N	A	‘muCHG’
36	Loewdin atomic charge	*q*_lo_	*e*	N	A	‘loCHG’
37	Mayer atomic charge	*q*_ma_	*e*	N	A	‘maCHG’
38	Atomic forces	*F*_at_	eV/Å	3N	A	‘vF’
39	Atomisation energy	*E*_at_	eV	1	M	‘eAT’

Property types are categorized into atomic (A) and molecular (M). *E*_h_and *a*_0_ refer to the atomic unit of Hatree and Bohr radius. * The number of orbital energies varies for each molecule.

### MORE-Q-G2 dataset generation: properties of the molecular dimers

The MORE-Q-G2 dataset contains the QM properties of the optimized structures of the 23,838 BOV-receptor systems (referred to as the dimer system). The configurations of the initial 1, 836 BOV-receptor systems (combination of 18 molecular receptors with 102 BOV molecules from MORE-Q-G1) were screened by molecular docking using the automated Interaction Site Screening (aISS)^60^ submodule of the xTB packages (version 6.6.0), where the GFN2-xTB parameterization and D4 dispersion correction were implemented. The aISS module prescreens potential docking sites (pockets, stack, and angular search) on the receptor, followed by a genetic optimization for stack and angular search, where the intermediate binding energies are evaluated using the xTB-IFF force field^72^. In the end, the updated dimer structures were optimized again by the GFN2-xTB method. To keep the same structural conformation of the receptor adsorbed on the graphene layer, we have fixed the geometry of the receptor during the docking process. Then, 100 configurations were generated per molecular dimer. We subsequently sent the dimer configurations back to the graphene surface and excluded those configurations in which any atom of the BOV molecule was located between the receptor and the graphene layer, resulting in a subset of 83,916 dimer configurations. It is worth mentioning that the atomic coordinates of the receptor were mapped exactly to the previous configuration on graphene from the MORE-Q-G1 dataset. As a final step, the hierarchical clustering method was used to filter similar geometries for each BOV-receptor combination, reducing the number of configurations up to 23,838. This step was carried out by computing the root-mean-square deviation (RMSD) among molecular structures. The detailed clustering process is discussed in Sec. 2 of the SI.

#### Calculation of physicochemical properties

We have first computed 24 QM global and local properties of the optimized structures of 23,838 BOV-receptor systems, see Table 3. These properties were obtained from the output files of a follow-up single-point calculation using GFN2-xTB, which considers D4 dispersion correction. The SCC convergence for these calculations was set to 1 ⋅ 10^−6^ *E*_h_. To name a few properties, we have energy components, orbital energies, *C*_6_ and *C*_8_ dispersion coefficients, atomic polarizabilities, dipole moment, quadrupole moment, and binding energy. Moreover, we have selected the most energy-favorable configuration for each dimer (ranked by the binding energy *E*_int_) and computed QM properties at the PBE+D3 level with def2-TZVPP basis set, as was previously done for the generation of MORE-Q-G1. Accordingly, MORE-Q-G2 also contains the 39 QM global and local properties listed in Table 2 and *E*_int_ for 1,836 dimers at PBE+D3 level.

**Table 3:** List of physicochemical properties of molecular dimers at GFN2-xTB+D4 theory level contained in MORE-Q-G2 subset.

#	Property	Symbol	Unit	Dimension	Type	HDF5 keys
1	Atomic number	—	—	N	A	‘atNUM’
2	Atomic positions	—	Å	3N	A	‘atXYZ’
3	Total GFN2-xTB+D4 energy	*E*_tot_	eV	1	M	‘eXTB+D4’
4	Repulsion energy	*E*_rep_	eV	1	M	‘eREP’
5	SCC total energy	*E*_scc_	eV	1	M	‘eSCC’
6	Isotropic electrostatic energy	*E*_iel_	eV	1	M	‘eIE’
7	Anisotropic electrostatic energy	*E*_ael_	eV	1	M	‘eAE’
8	Anisotropic exchange-correlation energy	*E*_axc_	eV	1	M	‘eAXC’
9	D4 dispersion energy	*E*_D4_	eV	1	M	‘eD4’
10	HOMO energy	*E*_HOMO_	eV	1	M	‘eH’
11	LUMO energy	*E*_LUMO_	eV	1	M	‘eL’
12	HOMO-LUMO gap	*E*_GAP_	eV	1	M	‘HLgap’
13	Orbital energies	*E*_oe_	eV	*	M	‘eORB’
14	Atomisation energy	*E*_at_	eV	1	M	‘eAT’
15	Atomic coordination number	*N*_ac_	—	N	A	‘ACN’
16	Atomic Mulliken charge	*q*_mu_	*e*	N	A	‘muCHG’
17	Atomic *C*_6_ dispersion coefficient	*C*_6,at_	${E}_{{\rm{h}}}\cdot {a}_{0}^{6}$	N	A	‘atC6’
18	Atomic polarizability	*α*_at_	${a}_{0}^{3}$	N	A	‘atPOL’
19	Isotropic *C*_6_ dispersion coefficient	*C*_6_	${E}_{{\rm{h}}}\cdot {a}_{0}^{6}$	1	M	‘mC6’
20	Isotropic *C*_8_ dispersion coefficient	*C*_8_	${E}_{{\rm{h}}}\cdot {a}_{0}^{8}$	1	M	‘mC8’
21	Isotropic molecular polarizability	*α*_s_	${a}_{0}^{3}$	1	M	‘mPOL’
22	Dipole moment	*μ*	*e* ⋅ *a*_0_	3	M	‘vDIP’
23	Scalar total dipole moment	*μ*_s_	D	1	M	‘DIP’
24	Molecular quadrupole tensor	*QP*	$e\cdot {a}_{0}^{2}$	6	M	‘QP’
25	Binding energy	*E*_int_	eV	1	M,BD	‘eBIND’

Each property presents a name, symbol, unit, dimension, type, and corresponding key in the HDF5 file. Property types are categorized into atomic (A) and molecular (M). BD stands for the binding feature. *E*_h_ and *a*_0_ refer to the atomic unit of Hatree and Bohr radius. For the most stable dimer conformation, we have also computed the same properties as listed in Table 2 at PBE+D3 level of theory, including the binding energy. *The number of orbital energies varies for each molecule.

### MORE-Q-G3 dataset generation: properties of the complex systems

The MORE-Q-G3 dataset contains the QM properties of the optimized structures of 1, 836 BOV-receptor-graphene systems (referred to as the complex (CPLX) system). To generate MORE-Q-G3, we have considered the most energy-favorable dimer configuration for each dimer (ranked by *E*_int_ from MORE-Q-G2) and, then, mapped it back to the graphene layer. Next, the CPLX systems underwent geometry optimization using the DFTB+ package, employing the GFN2-xTB Hamiltonian with D4 dispersion correction for the SUB system. We here chose to fix the atomic positions in the graphene layer, as the adsorption of BOV molecules will not significantly affect them. The resulting 1,836 CPLX systems were then split into SUB systems and BOV molecules (OM) to compute binding features.

#### Calculation of physicochemical properties

We have first computed 20 QM global and local properties of the optimized structures of 1,836 CPLX systems, 1,836 SUB systems, and 1,836 OM systems, see the top panel in Table 4. In doing so, single-point calculations of these systems were conducted at tightly converged PBE+D3 theory level by Vienna ab initio simulation package^73,74^ (VASP, version 6.3.1). The energy cutoff for the plane-wave basis set and the SCF convergence threshold were set to 600 and 1 ⋅ 10^−5^ eV, respectively. And all simulations were conducted at Gamma point. The dipole correction along the slab direction (50.68 Å) was switched on to obtain flat electrostatic potential in the slab. Energy components, orbital energies, atomic forces, stress tensor, and electrostatic potential were extracted from the OUTCAR output file. Bader atomic charges *q* were obtained by postprocessing the information obtained from Bader charge analysis^75^.

**Table 4:** List of physicochemical properties of molecular systems at PBE+D3 theory level contained in MORE-Q-G3 subset.

#	Property	Symbol	Unit	Dimension	Type	HDF5 keys
1	Atomic number	—	—	N	A,S	‘atNUM’
2	Atomic coordinates	—	Å	3N	A,S	‘atXYZ’
3	Total PBE+D3 energy	*E*_tot_	eV	1	G,S	‘ePBE+D3’
4	Fermi energy	*E*_F_	eV	1	G,S	‘eFE’
5	E6 dispersion energy	*E*_6_	eV	1	G,S	‘eE6’
6	E8 dispersion energy	*E*_8_	eV	1	G,S	‘eE8’
7	Total dispersion energy	*E*_D3_	eV	1	G,S	‘eD3’
8	Valence band maximum	*E*_vbm_	eV	1	G,S	‘eVBM’
9	Conduction band minimum	*E*_cbm_	eV	1	G,S	‘eCBM’
10	VBM-CBM gap	*E*_gap_	eV	1	G,S	‘eGAP’
11	Band energies	*E*_be_	eV	*	G,S	‘eBE’
12	Work function	*ϕ*	eV	1	G,S	‘WF’
13	Planar-averaged potential z distance	*z*	Å	*	G,S	‘zEPOL’
14	Planar-averaged potential	*P*_avg_	eV	*	G,S	‘eEPOL’
15	Cell parameters	*l*	Å	9	G,S	‘CELL’
16	Cell stress tensor	*σ*	kB	6	G,S	‘stCELL’
17	External cell pressure	*P*_cl_	kB	1	G,S	‘pCELL’
18	Atomic forces	*F*_at_	eV/Å	3N	A,S	‘vF’
19	Total drift	*F*_df_	eV/Å	3	A,S	‘vDF’
20	Bader atomic charge	*q*	*e*	N	A,S	‘baCHG’
1	Adsorption energy	*E*_ads_	eV	1	G,BD	‘eADS’
2	Graphene Bader charge change	Δ*Q*_GR_	*e*	1	G,BD	‘GbaDCHG’
3	Receptor Bader charge change	Δ*Q*_rec_	*e*	1	G,BD	‘RbaDCHG’
4	BOV molecule charge change by Bader analysis	Δ*Q*_om_	*e*	1	G,BD	‘ObaDCHG’
5	Work function change	Δ*ϕ*	eV	1	G,BD	‘DWF’
6	Dispersion energy change	Δ*E*_D3_	eV	1	G,BD	‘DD3’
7	Electronic gap change	Δ*E*_gap_	eV	1	G,BD	‘DGAP’
8	Bader atomic charge change	Δ_*q*_	*e*	*	A,BD	‘baDCHG’

Each property presents a name, symbol, unit, dimension, type, and corresponding key in the HDF5 file. Property types are categorized into atomic (A) and global (G). S and BD stand for a single system (*e.g*. CPLX, SUB, and OM) and for the binding feature, respectively. *E*_h_ and *a*_0_ refer to the atomic unit of Hatree and Bohr radius. *The dimension of these properties varies for each molecule.

The work function of the CPLX and SUB systems were calculated as follows:

1 $$\phi ={E}_{{\rm{V}}}-{E}_{{\rm{F}}},$$

where *E*_F_ is the Fermi level and *E*_V_ is the vacuum energy. And the work function change is defined as the work function difference after and before the BOV adsorption *i.e*. CPLX and SUB systems:

2 $$\Delta \phi ={\phi }_{{\rm{CPLX}}}-{\phi }_{{\rm{SUB}}}.$$

*E*_V_ is obtained by analyzing the flattened region of the electrostatic potential *P*(*z*) along the slab direction. *P*(*z*) is computed by the following equation:

3 $$P(z)=\int \,n(z)dz,$$

where the planar averaged charge density *n*(*z*) is defined as:

4 $$n(z)=1/A\iint \,n(x,y,z)dxdy$$

and the *A* denotes the surface area of the cell.

To investigate the sensitivity and selectivity of the receptors, we have also calculated 8 binding features for these systems, see the bottom panel in Table 4. For example, the adsorption energy *E*_ads_, which is defined as the interaction strength between OM and SUB systems, was computed by evaluating:

5 $${E}_{{\rm{ads}}}={E}_{{\rm{CPLX}}}-{E}_{{\rm{SUB}}}-{E}_{{\rm{OM}}},$$

where *E*_CPLX_, *E*_SUB_, and *E*_OM_ are the total energy of each system obtained by VASP. The atomic charge change Δ*q* is obtained by the difference between the Bader atomic charge *q* of the same atom in CPLX and SUB (OM) systems. By summing up the atomic charge changes of the atoms for individual components, we can obtain the charge change for the receptor (Δ*Q*_rec_), BOV molecule (Δ*Q*_om_), and graphene substrate (Δ*Q*_GR_) upon adsorption of BOV molecules. The other binding features were computed similarly, taking into account the values obtained for the CPLX and SUB systems.

### Interconnection between the MORE-Q subsets

Here, we summarize the interactions among the three MORE-Q subsets in the following points:

- The MORE-Q-G1 subset contains QM property data for 102 BOV molecules and 18 molecular receptors. Among the 39 molecular and atomic properties, we computed the D3 energy, dipole moment, polarizability, and Mulliken charges (see the property list in Table 2).
- The MORE-Q-G2 subset is built on the geometries from MORE-Q-G1 via the search for molecular docking conformations using BOV molecules and receptors. Accordingly, MORE-Q-G2 contains QM property data for 23,838 dimer conformations at the GFN2-xTB+D4 level and for 1,836 dimers with the lowest binding energies at the PBE+D3 level (see the property list in Tables 2 and 3).
- The MORE-Q-G3 subset is constructed by depositing 1,836 selected dimers from MORE-Q-G2 onto a graphene surface. Consequently, MORE-Q-G3 includes QM property data at the PBE+D3 level for both the CPLX and SUB systems, as well as binding features that account for property changes in single systems induced by BOV molecule adsorption (see the property list in Table 4).

## Data Records

The MORE-Q datasets are available in three HDF5 files in the ZENODO.ORG data repository^76^. Indeed, one can find there the files MORE-Q-G1.hdf5, MORE-Q-G2.hdf5, and MORE-Q-G3.hdf5 corresponding to the three MORE-Q datasets described in this work. We also provide a README file with technical usage details and examples of how to extract data from the HDF5 files and, then, convert it to Python pandas Dataframes for further analysis (see createDF.py file)

### HDF5 file format

#### File sutrcture

**Fig. 3:**
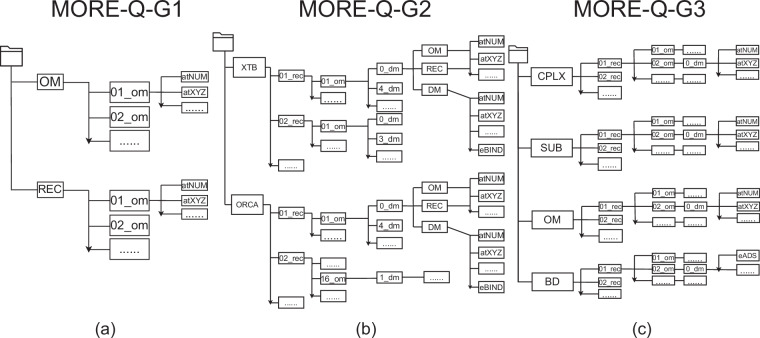
Architecture of the HDF5 files corresponding to (**a**) MORE-Q-G1, (**b**) MORE-Q-G2, and (**c**) MORE-Q-G3 subsets.

Independent of the MORE-Q subset, the information for each molecular structure is stored in a Python dictionary (dict) type containing all relevant properties and recorded in *groups* in HDF5 file format. The HDF5 file architecture of the MORE-Q subsets is depicted in Fig. 3.

- For MORE-Q-G1.hdf5 file, containing QM properties of BOV molecules (om) and molecular receptors (rec), nn_om and mm_rec are allocated to the main *groups* keys, where nn and mm range from 1 to 102 and from 1 to 18, respectively. HDF5 keys to access the atomic numbers, atomic positions (coordinates), and physicochemical properties in each dictionary are provided in Table 3.
- For MORE-Q-G2.hdf5 file, containing QM properties of the molecular dimers at different levels pf theory, XTB and ORCA become the main *groups* keys. Under both of them, mm_rec is created as the subgroup. Then, a nn_om subgroup is created per mm_rec subgroup, where nn_om and mm_rec represent the combination of BOV molecules (om) and molecular receptors (rec) that compose a given molecular dimer. Following this, a third-level subgroup, ll_dm, is created to indicate the dimer configurations within each nn_om subgroup, where ll denotes the order of the dimers. Under the ll_dm subgroup, DM (dimer), OM (isolated BOV molecule), and REC (receptor) subgroups were created to store the QM properties listed in Tables 3 and 2 for each system. The binding energy values are saved in the DM subgroup.
- For MORE-Q-G3.hdf5 file, containing QM properties of the complex systems (*i.e*. BOV-receptor-graphene), substrate systems (*i.e*. receptor-graphene), BOV molecules, and the binding features, the main *groups* keys CPLX, SUB, OM and BD are constructed. Alike the structure of MORE-Q-G2.hdf5 file, we have first created the mm_rec subgroup for each main group. Then, the subgroup, nn_om, is created per nn_om subgroup, representing the BOV molecular and molecular receptor combination. Followed by ll_dm, the dimer configuration order is indicated. QM properties listed in Table 4 are then stored per subgroup.

#### Property format

MORE-Q HDF5 files contain various types of data derived from the features of each property. The atomic numbers are stored as a list of strings, with each string representing the atomic number of a corresponding atom in the molecule. The atomic coordinates and forces are saved as a list of 3N vectors, where each vector contains the x, y, and z components. Orbital (band) energies are saved as a vector array, containing 10 energies below HOMO (VBM) level and 10 energies above the LUMO (CBM) level. Vectorial and tensorial properties are also saved as a vector array, with the order maintained as x, y, z for vectorial properties and xx, yy, zz, xy, yz, zx for tensorial properties. For all atomic properties, the order of the vector array is identical to the atomic coordinates vector. For the MORE-Q-G2 and MORE-Q-G3 subsets, the order of the atom type follows receptor → BOV and graphene → receptor → BOV, respectively. This order is also applicable to every atomic property. The other properties are single values that could be directly called by the corresponding HDF5 keys, see Tables 2–4.

## Technical Validation

Unlike other datasets in the field of digital olfaction, the MORE-Q datasets contain an extensive set of quantum-mechanical (QM) properties for the building blocks of graphene-based molecular sensors, as well as binding features among the sensor components (see Fig. 2). Indeed, structural properties were obtained using the semi-empirical GFN2-xTB method with D4 dispersion correction^63^, which is known to generate correct geometries at an efficient computational cost. While the energetic, atomic forces and other property calculations were performed at the more accurate level of theory such as PBE with D3 dispersion correction^77^, as implemented in the VASP code. By doing this, we have guaranteed both sufficiently accurate QM properties and feasible computational time consumption.

Before constructing our complex CPLX system (*i.e*. BOV-receptor-graphene system), we have determined the optimal configuration for the molecular receptor adsorbed on the graphene layer. In this regard, it is known that *π* − *π* stacking interaction is the main functionalization mechanism of the mucin-derived receptor. Accordingly, the receptor configuration with the maximal pyrene rings interacting with graphene will be the most stable receptor-graphene system (referred to as the SUB system). Based on this concept, we initially deposited each receptor on graphene in up to four configurations with diverse orientations, which were subsequently optimized using GFN2-xTB methods with D4 correction and tight settings of convergence. Then, the most stable configurations with the lowest adsorption energy were taken as the backbone structures, which were used to further generate the MORE-Q datasets, as shown in Fig. 2. Note that a more exhaustive evaluation of the conformational space of the molecular receptors may yield different results. However, the main focus of the current work is to define the pathways for understanding and predicting QM-based structure-property and property-property relationships in potential molecular sensors for digital olfaction. Thus, the selection of these configurations represents the most likely configurations and, therefore, ensures the baseline quality of the dataset.

**Fig. 4:**
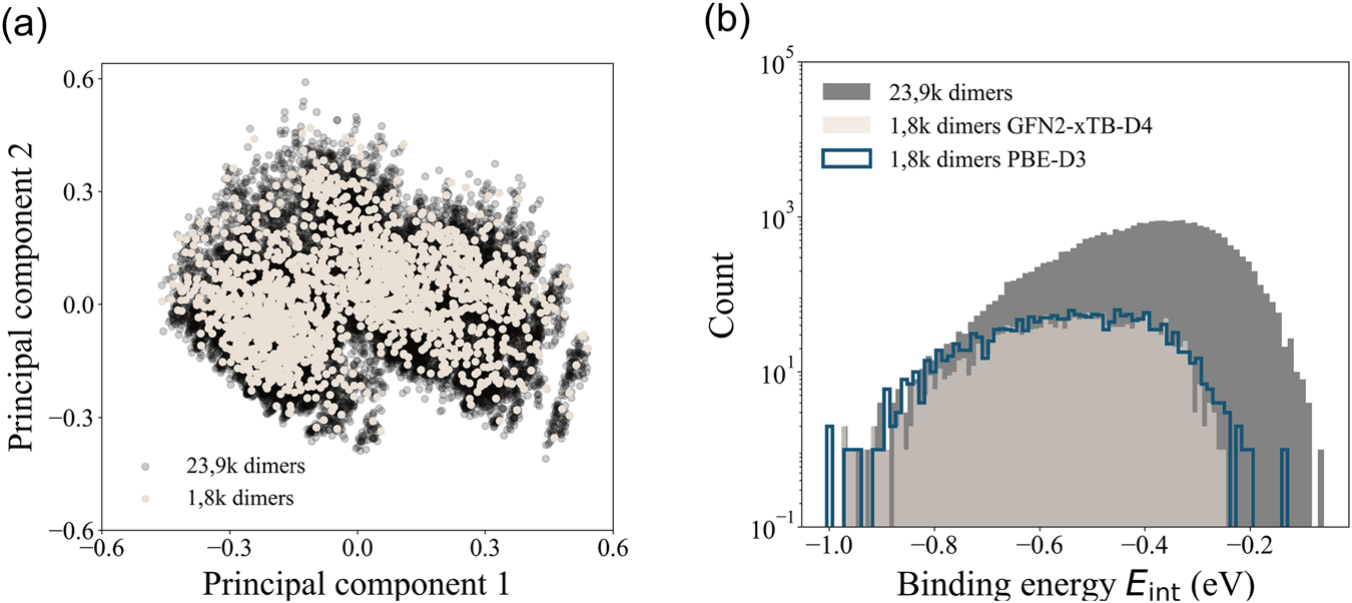
Analysis of the conformational and energetic space for molecular dimers in MORE-Q-G2 subset. (**a**) Two-component principal component analysis based on the global properties for the 23,838 (black circles) dimer conformations stored in MORE-Q-G2. For comparison, we show the values corresponding to the 1,836 most energetically stable dimer conformation (white circles). (**b**) Frequency plots for the GFN2-xTB+D4 binding energies of both molecular subsets discussed in panel (**a**). We also show the plot corresponding to the energy values obtained using PBE+D3 level of theory for the 1,836 most energetically stable dimer conformation (blue silhouette).

Next, the selected receptor configurations were combined with BOV molecules to form the input geometries for the aISS code^60^ to determine the docking sites. This method has been successfully used to determine the explicitly solvated structures of peptides and macrocycles from the MPCONF196 dataset^78^. After performing the docking procedure, hierarchical clustering^79^ based on root-mean-squared deviation (RMSD) and energetics was carried out to select non-redundant configurations, resulting in a total of 23,838 molecular dimers (see more details for clustering in Fig. S3 of the SI). From this subset, we have selected the most energy-favorable configuration per molecular dimer (ranked by the binding energy *E*_int_) for further examination. Indeed, the principal component analysis (PCA) is conducted on the 23,838 and the selected 1,836 configurations using the global properties stored in MORE-Q-G2 dataset. Fig. 4(a) shows the PCA space for both molecular sets, where one can see that the selected 1, 836 configurations span the entire region covered by the 23,838 dimers. This indicates that the reduced set is a representative sample of the dimer configurations. As shown in Fig. 4(b), even though we sampled only configurations with the lowest *E*_int_, the coverage of the initial *E*_int_ values is considerable when considering the reduced molecular set. Here, configurations with $\left|{E}_{{\rm{int}}}\right|$ smaller than 0.25 eV were filtered out after the energetic selection, as these meta-stable dimers exhibit weak non-covalent interactions and are unlikely to occur during the docking process — another compelling evidence that the reduced set is a representative sample. Additionally, we computed *E*_int_ at the PBE+D3 level of theory for the 1,836 dimers, and the corresponding values were very similar (see plot with blue solid lines in Fig. 4(b)).

**Fig. 5:**
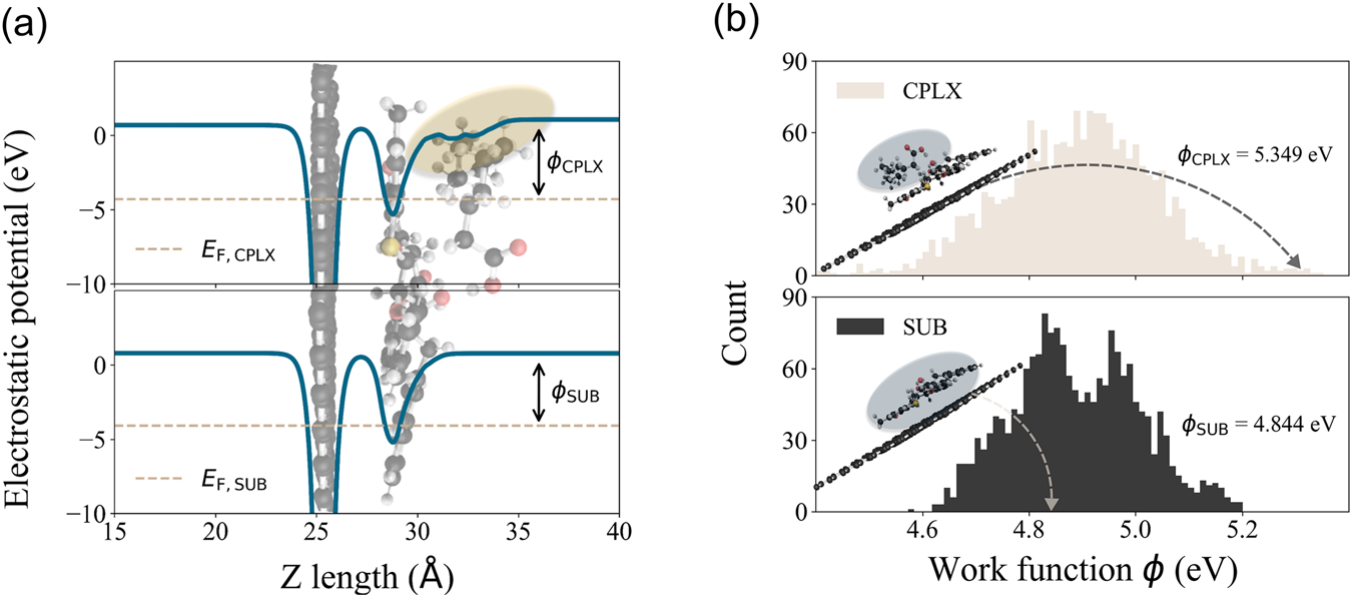
Understanding work function *ϕ* calculations of molecular systems in MORE-Q-G3. (**a**) Example case of the system including BOV-35 and Receptor-18: planar-average electrostatic potential along the slab length (*z* direction) for the CPLX and SUB systems (darkblue curve) with their corresponding Fermi level (dashed line). The arrows for *ϕ*_CPLX_ and *ϕ*_SUB_ denote the work function values for each system. The geometry of the CPLX system was placed to correlate the atomic positions with the potential. The change in the potential curve originated from the adsorption of BOV molecule is highlighted by the orange shadow. (**b**) Frequency plots for the work function values of the 1,836 CPLX (white) and SUB (black) systems. The *ϕ* value for the inserted conformations of CPLX and SUB systems are 5.35 eV and 4.84 eV, respectively.

Another unique feature of the MORE-Q dataset is the calculation of the work function, *ϕ*, for the SUB and CPLX systems (see Eqs. (1)~(4) in “Methods” section). *ϕ* and the change in work function upon adsorption, Δ*ϕ*, are relevant factors in evaluating the sensing performance *via* the analysis of the electronic responses^80–86^. Indeed, Δ*ϕ* has already been used as an indicator for the detection of halogen ions in self-assembled alkylammonium halide monolayer-modified substrates^87^. Li *et al*.^88^ also conducted systematic density functional theory (DFT) calculations on ten small molecules on the WO_3_ substrate, evaluating their sensitivity and selectivity using Δ*ϕ*. Similarly, recent computational studies have highlighted the critical role of work function calculations in sensor applications^89–95^, underscoring the significance of this electronic property in molecular sensing. Thus, in our work, *ϕ* for SUB and CPLX systems was calculated with a careful treatment of the slab thickness and dipole correction, which ensures the smooth and flat region of the electrostatic potential in vacuum *E*_V_, see Fig. 5(a). *ϕ* is then defined as the energy difference between *E*_V_ and Fermi level *E*_F_, see Eq. (1). In the top panel of Fig. 5(a), we show an example of the effect of the adsorption of the BOV molecule on the electrostatic potential of the SUB system (see orange shadow). This alteration of the potential can result in an increase or decrease in *ϕ*, a magnitude that will be defined as Δ*ϕ*. Figure 5(b) presents the frequency plots of *ϕ* for SUB and CPLX systems. Here, we observe that the functionalization of the receptor on graphene spreads *ϕ* in the range [4.6, 5.2] eV, with the highest concentration of *ϕ* values in the range [4.8, 5.0] eV. With the adsorption of BOV molecules onto the SUB systems to form the CPLX system, the bimodal-like distribution transforms into a normal-like distribution, with *ϕ* values spreading more broadly over the range [4.4, 5.4] eV. This exemplifies the effect of BOV-receptor intermolecular interactions and illustrates the pivotal role of *ϕ* in understanding the sensing performance of these molecular systems. As shown above, the generation of the MORE-Q dataset involved a series of computational tasks, including GFN-xTB+D4 geometry optimizations, molecular docking conformational searches, and electronic property calculations for both periodic and gas-phase systems. Out of approximately 9 million CPU hours spent performing these calculations, the work function calculations for the 1,836 CPLX and SUB systems were the most computationally expensive, accounting for circa 5 million CPU hours. All calculations were performed on CPUs with Intel Xeon Platinum 8470 processors.

**Fig. 6:**
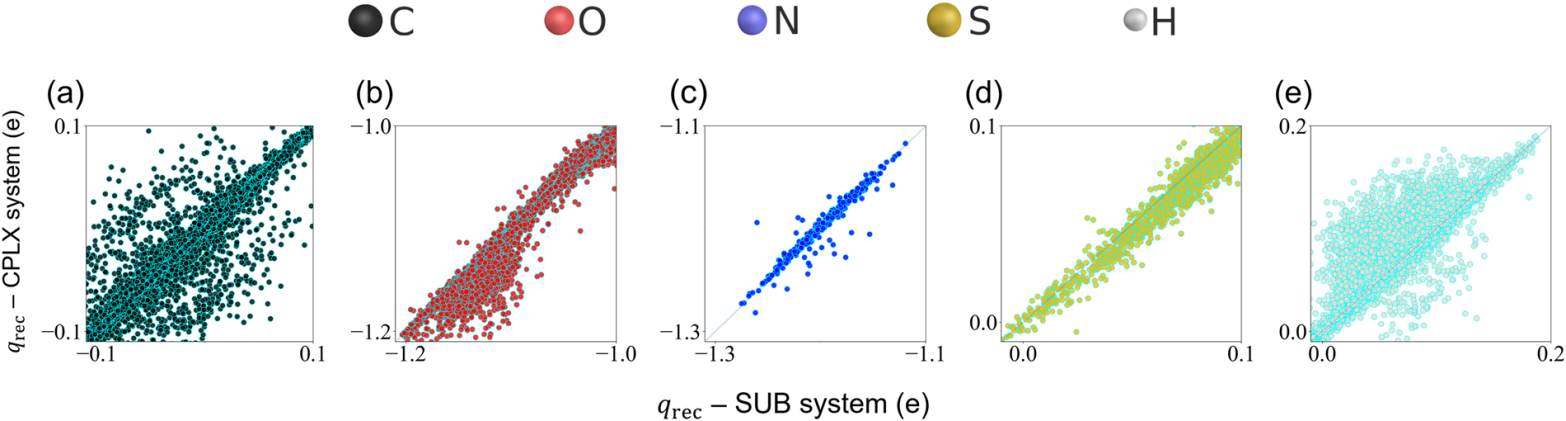
Correlation plots of the atomic charges in the molecular receptors *q*_rec_ of CPLX and SUB systems contained in MORE-Q-G3. From (**a**)→(**e**), one can see how atomic charges of Carbon, Oxygen, Nitrogen, Sulfur and Hydrogen atoms in the molecular receptor are modified by the adsorption of BOV molecule.

The atomic charges, *q*, of molecular receptors can also provide insights into sensing performance by examining how the adsorption of BOV molecules (*i.e*. CPLX system) affects the charge distribution in the molecular receptors. As shown in Fig. 6, the atomic charges of the C, H, and O atoms in the receptors are more affected by the BOV-receptor interaction compared to corresponding values of S and N atoms. Moreover, the increase and decrease in (positive/negative) *q* values reveal the existence of multiple charge transfer processes at reactive atomic sites in the receptors. These changes in charge distribution imply that the local chemical environments in the receptors have been modified — a result that can be further exploited to analyze the sensing mechanisms in these systems.

**Fig. 7:**
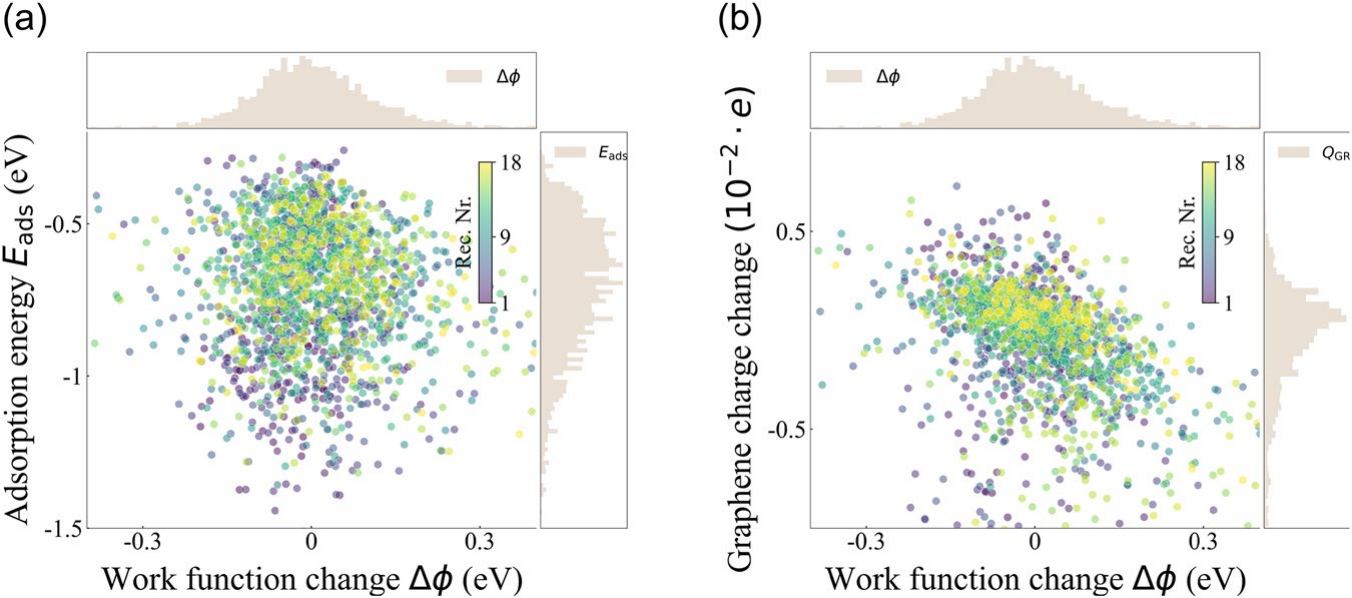
Validating the change of binding features in systems contained in MORE-Q-G3. Correlation plots between (**a**) adsorption energy *E*_ads_ and work function change Δ*ϕ*, and (**b**) Graphene charge change and work function change Δ*ϕ*. The corresponding frequency plot per binding feature is also plotted on each panel. For both panels, the datapoints are colored by the receptor number, see the inserted color bar.

The change of property values between CPLX and SUB systems is defined as binding features in the present work (see details in “Methods” section). Binding features serve as crucial indicators for the sensing response of SUB systems toward the adsorption of BOV molecules. Figure 7 depicts the correlation plots between select binding features: adsorption energy *E*_ads_ (see Eq. (5)), graphene charge change Δ*Q*_GR_, and work function change Δ*ϕ*. In Fig. 7(a), it can be seen that *E*_ads_ and Δ*ϕ* are uncorrelated, varying from  −0.3 to  −1.5 eV and from  −0.3 to 0.3 eV, respectively. Similarly, the change in the atomic charges of the graphene layer does not show any correlation with Δ*ϕ* (see Figure 7(b)). The small magnitude of the charge changes on graphene (−0.005 to 0.005 *e*) confirms the weak adsorption of the BOV molecules, while the atomic charge analysis on molecular receptors demonstrates their crucial role in the sensing workforce (*vide supra*). In both panels, the data points are colored according to the receptor number, indicating a lack of clustering based on the receptor. Thus, the “freedom of design” principle^96^ can also be applied to the MORE-Q dataset to gain a better understanding of structure-property and property-property relationships in these potential olfactory sensors.

Since MORE-Q is the first extensive QM dataset for molecular olfactorial receptor engineering, it is expected to have certain limitations. For example, MORE-Q currently spans the chemical space defined by BOV-receptor systems containing only C, H, O, N, and S atoms. To further expand its scope of applicability to more complex BOV molecules related to human body, atom types such as B, F, P, Se, Si, and Ge should be considered^6^. This expansion would also broaden the chemical design space for molecular receptors. Another crucial improvement for MORE-Q involves the inclusion of additional conformations of BOV-receptor systems in the calculations of binding features to construct MORE-Q-G3. This would provide a better understanding of the conformational effects on the olfactory response of molecular receptors. Finally, the computational accuracy of binding feature calculations could be enhanced by using more robust QM methods (*e.g*. hybrid functionals) that include van der Waals corrections, such as D4 or many-body dispersion. However, these improvements would significantly increase computational costs, potentially limiting the number of conformations that can be studied.

In summary, the MORE-Q dataset provides an opportunity to accurately investigate the sensing mechanisms of diverse BOV-receptor systems from a QM perspective. Besides global and local QM properties of monomers and dimer configurations, MORE-Q focuses on relevant binding features such as adsorption energy, charge transfer, and work function changes on graphene substrates. This exhaustive set of QM properties has the potential to enhance the fundamental understanding of the adsorption behavior of BOV molecules. Indeed, by leveraging this knowledge, one can develop robust and transferable machine learning models to predict binding features, which enable a rapid evaluation of sensing performance in molecular systems. MORE-Q can also facilitate the optimization and design of novel mucin-derived molecular receptors through the combination of computed QM property data and generative models, moving closer to the goal of developing biomimetic electronic noses. Additionally, integrating MORE-Q into perception studies of BOV molecules by linking the binding feature space with the human perceptual rating space has the potential to offer valuable insights into the cognitive processes underlying human olfaction.

## Supplementary information

Supplementary Information

## Data Availability

The initial BOV information processing of the screening procedure was conducted mainly using RDkit 2023.09.5^97,98^. Structural optimization has been conducted in the xTB version 6.6.0^59^ and DFTB+^67^ codes. The configuration search process was performed by the aISS program^60^. The planar-average potential for work function calculation was obtained by Vaspkit^99^. The PBE+D3 property calculation was conducted in ORCA version 5.0.3^68^ and VASP version 6.3.1^73,74^ packages together with ASE^100^. A user guide for reading and exploring different uses of the MORE-Q dataset has been added to the GitHub repository Repo-MORE-Q^101^.
