# Supplementary material for: MORE-Q, a dataset for molecular olfactorial receptor engineering by quantum mechanics
Source: Sci Data. 2025 Feb 22;12:324. doi: 10.1038/s41597-025-04616-6 (PMC11846975; doi:10.1038/s41597-025-04616-6)
Supplement: Supplementary file 1 — Supplementary Information [file 41597_2025_4616_MOESM1_ESM.pdf]

SUPPORTING INFORMATION FOR MORE-Q, A DATASET FOR MOLECULAR OLFACTORIAL RECEPTOR ENGINEERING BY QUANTUM MECHANICS

Li Chen[1], Leonardo Medrano Sandonas[1,*], Philipp Traber[3], Arezoo Dianat[1], Nina Tverdokhleb[1], Mattan Hurevich[4], Shlomo Yitzchaik[4], Rafael Gutierrez[1], Alexander Croy[3,*], and Gianaurelio Cuniberti[1,2,*]

[1]*Institute for Materials Science and Max Bergmann Center for Biomaterials, TUD Dresden University of Technology, 01062 Dresden, Germany*

[2]*Dresden Center for Computational Materials Science (DCMS), TUD Dresden University of Technology, 01062 Dresden, Germany*

[3]*Institute of Physical Chemistry, Friedrich Schiller University Jena, 07737 Jena, Germany*

[4]*Institute of Chemistry and Center of Nanotechnology, The Hebrew University of Jerusalem, Jerusalem 91904, Israel*

[*]corresponding authors: Leonardo Medrano Sandonas (leonardo.medrano@tu-dresden.de), Alexander Croy (alexander.croy@uni-jena.de), Gianaurelio Cuniberti (gianaurelio.cuniberti@tu-dresden.de)

1 Body odor molecules screening

The first publication is a topical review from Drabińska et al. [1] about the so-called human volatilome. It reports a meta-analysis of the available literature on where in the human body chemical substances have been documented, which are categorized into feces, urine, breath, skin, milk, blood, saliva and semen. This dataset contains information for all structures via their CAS number, compound name, sum formula, and their respective literature source for the categories "feces", "urine", "breath", "skin", "milk", "blood", "saliva" and "semen". Next, we ensure that all compounds have a valid CAS number, or exclude entries without one, to only feature distinct substances. We then take the CAS numbers and added the respective PubChem compound ID (CID) to the data frame using the pubchempy package. Next, we dropped all data without a CID number, as no further data on these substances can be downloaded consistently. For the remaining structures, we added all data from PubChem based on the mentioned API by constraining the heavy atoms to C, O, S, and N. Further, we chose to focus on the skin subgroup of the remaining molecules, as we will be dealing with odor molecules found in sweat. A central aspect of the project is the perception of odor molecules and, therefore, we added perception ratings to the molecules as an additional dimension. In 2016, Keller et al. [2] published a study, presenting a big survey involving 480 molecules and their perception. We took the intersection of the two molecule sets based on their CID numbers and ended up with 102 skin-related molecules with perception data. The 102 BOV molecules overview is shown in Fig. S1.

2 Mucin-derived receptor

The 18 mucin-derived receptors are composed of glycans modified by aromatic decoration for surface adhesion. Glycans are critical in signal transductions in biological systems. This signal is triggered by significant morphological and density changes during recognition events. Many glycans are located at the air-water interface, they interact with various entities, including microorganisms, food, and airborne molecules. The modification and structure of glycoconjugates are crucial in the functioning of the respiratory and olfactory systems. Integrating glycans into electronic and optoelectronic systems presents a transformative opportunity in sensor technology. By utilizing the unique chemical properties of glycans in hybrid systems, we designed sensors with enhanced sensitivity and specificity. Our goal is to mimic and utilize the responsiveness and functionality of glycans in the air-water environment, creating selective odor-reactive surfaces for integration with hybrid 2D materials-based devices. D-galactose, one of the most common glycans in the extracellular matrix, was used as a scaffold for aromatic decorated monosaccharide receptor's library. A strategy for multistep chemical synthesis [3] of monosaccharides decorated with various aromatic groups presents a key step toward obtaining this library. The synthetic approach provides the ability to install specific groups of various natures on the monosaccharide thereby enabling tuning the receptor's affinity toward odorants. Using this ability we control the rigidity, hydrophobicity and the polarizability of glycan-based receptors.

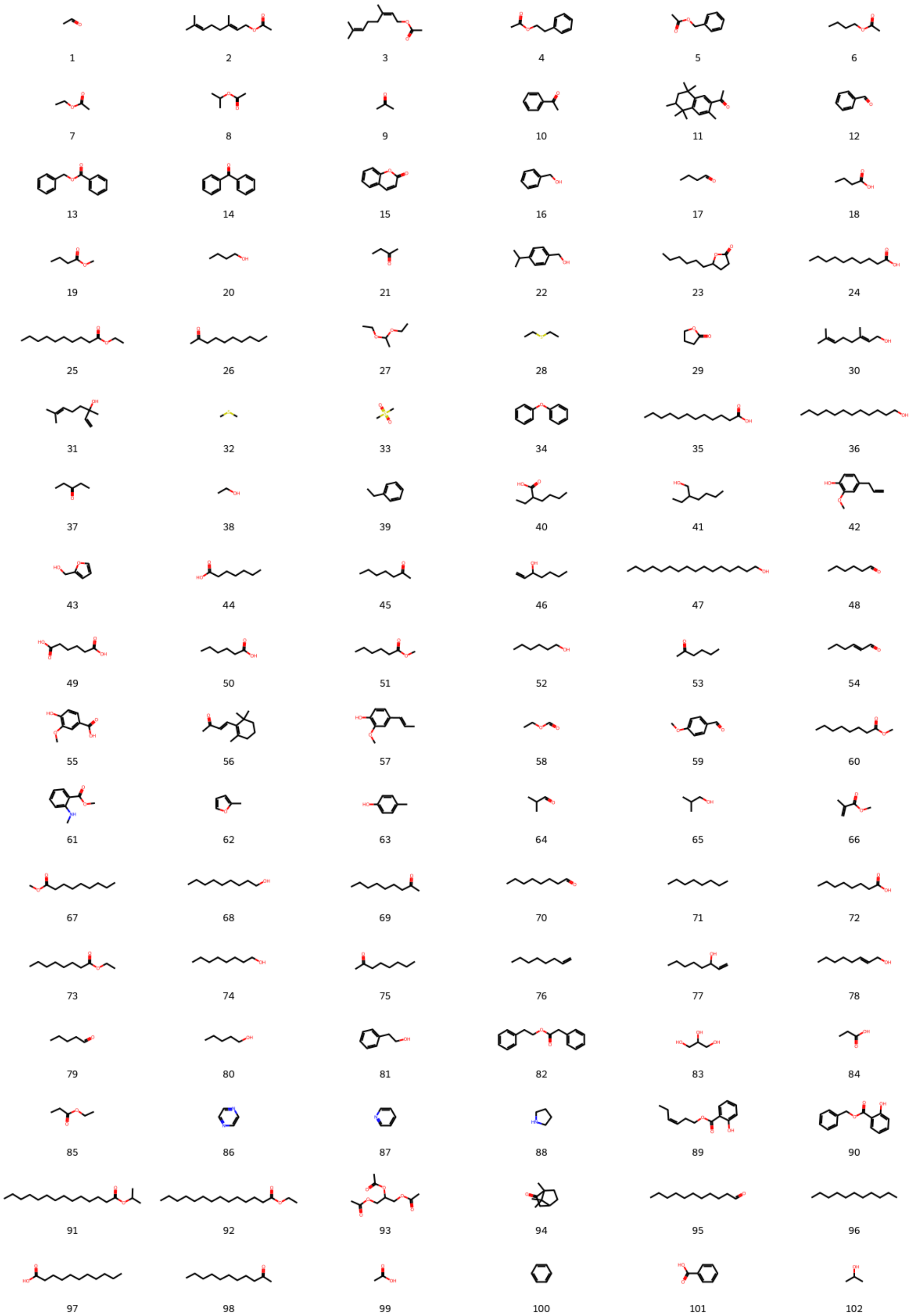

Figure S1: The 102 BOV molecules overview

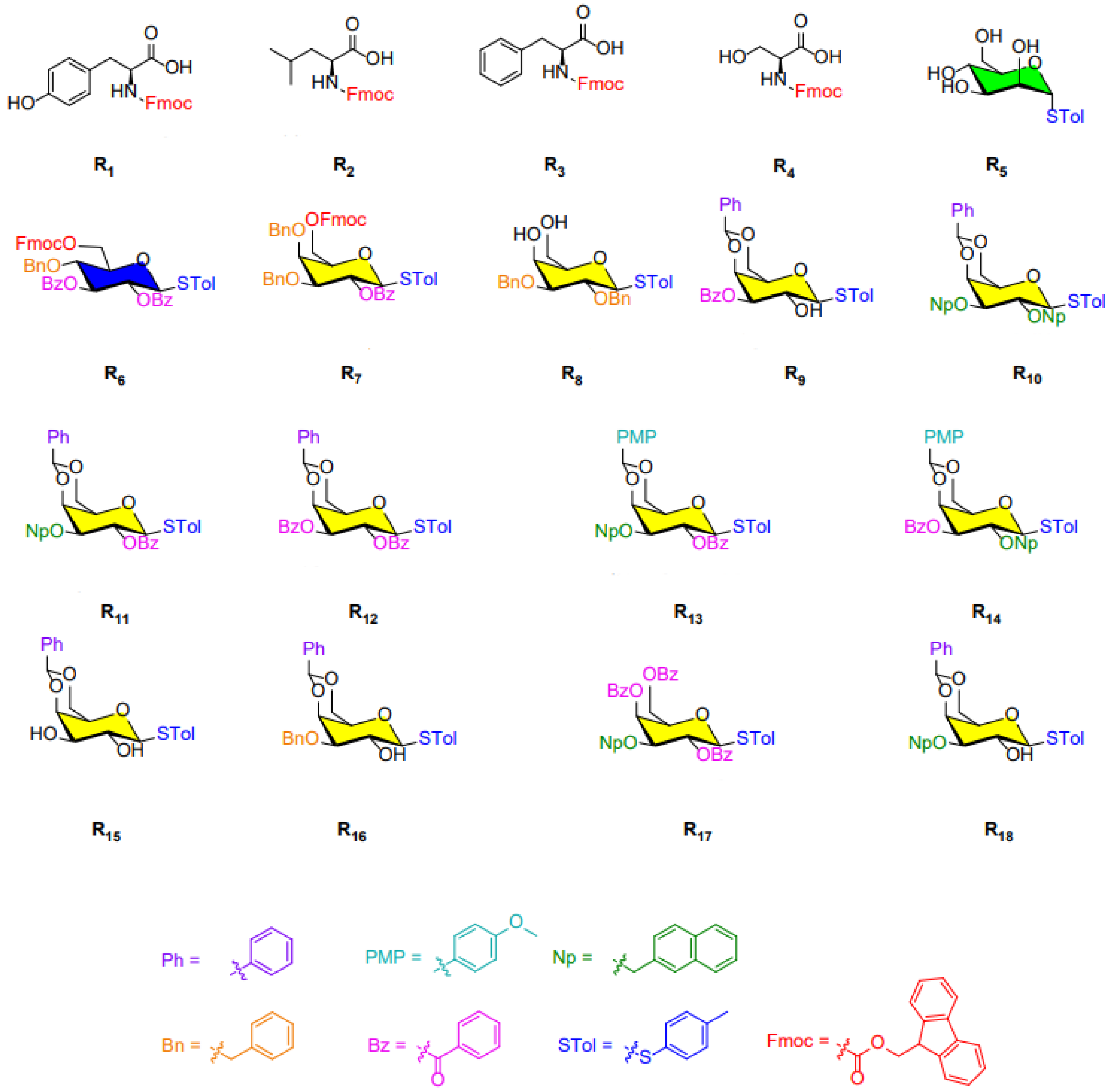

Figure S2: Chemical structure of the 18 receptors (R1∼R18). Aromatic functional groups are depicted below the receptors

3 Hierarchical clustering methods for the $83,916$ docking configurations

As shown in Fig. 1, a root-mean-square deviation (RMSD) -based hierarchical clustering method was implemented to select the non-redundant $23,838$ from the $83,916$ docking configurations. Fig. S3 (a) shows the cluster number concerning the cut-off similarity distance variance. Each boxplot contains the cluster number of the $1,836$ BOV-receptor combination. And the red line connects the cluster number median value for each similarity distance. (b) $\sim$ (d) show the cluster number distribution of these $1,836$ combinations. To ensure an efficient computation time as well as an exhaustive configuration search, the 1.5Å cut-off distance was taken, which led to $23,838$ configurations for MORE-Q-G2.

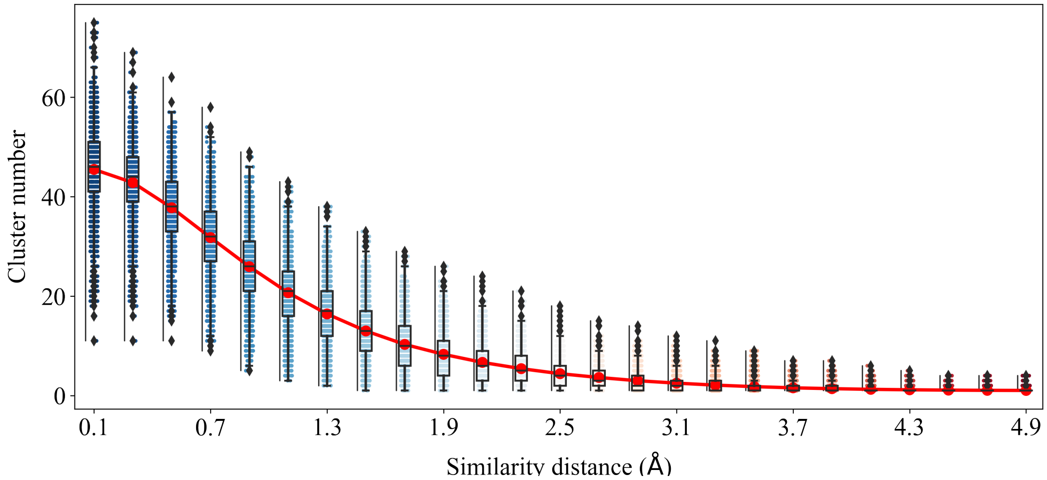

(a)

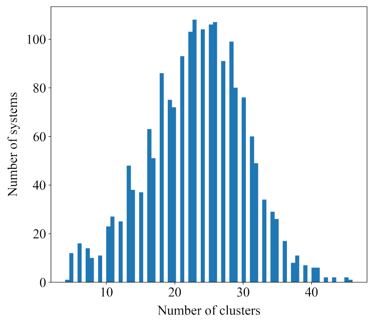

(b) rmsd=1Å

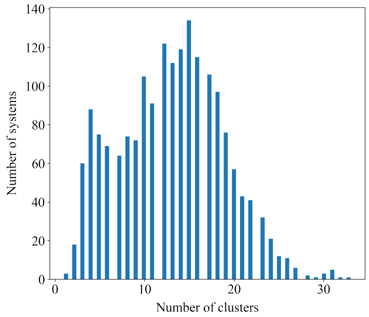

(c) rmsd=1.5Å

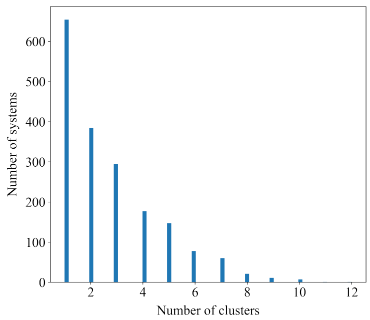

(d) rmsd=3Å

Figure S3: (a) Similarity distance convergence. Red line is the connection of the cluster number median value. (b) $\sim$ (d) are the cluster number distribution over the $1,836$ BOV-receptor combinations.

4 Additional information of properties calculation

4.1 Inertia moment

The inertia moment tensor components were calculated by $I_{xx} = \Sigma m_i(y_i^2 + z_i^2)$, $I_{yy} = \Sigma m_i(x_i^2 + z_i^2)$, $I_{zz} = \Sigma m_i(x_i^2 + y_i^2)$, $I_{xy} = I_{yx} = -\Sigma m_i y_i z_i$, $I_{xz} = I_{zx} = -\Sigma m_i x_i z_i$, and $I_{yz} = I_{zy} = -\Sigma m_i y_i z_i$, where x_i, y_i, and z_i specifies the ith atom position components.

4.2 Multi-dimensional property direction rule in MORE-Q

tensor vector direction order: XX YY ZZ XY XZ YZ

Vector direction order: X Y Z

Cell vector direction: X (x,y,z) Y (x,y,z) Z (x,y,z)

References

[1] Natalia Drabińska, Cheryl Flynn, Norman Ratcliffe, Ilaria Belluomo, Antonis Myridakis, Oliver Gould, Matteo Fois, Amy Smart, Terry Devine, and Ben De Lacy Costello. A literature survey of all volatiles from healthy human breath and bodily fluids: the human volatilome. *Journal of breath research*, 15(3):034001, 2021.

[2] Andreas Keller and Leslie B Vosshall. Olfactory perception of chemically diverse molecules. *BMC neuroscience*, 17:1–17, 2016.

[3] Yonatan Sukhran, Israel Alshanski, Ofer Filiba, Megan J. Mackintosh, Igor Schapiro, and Mattan Hurevich. Unexpected nucleophile masking in acyl transfer to sterically crowded and conformationally restricted galactosides. *The Journal of Organic Chemistry*, 88(13):9313–9320, June 2023.
